# Infectious Schmallenberg Virus from Bovine Semen, Germany

**DOI:** 10.3201/eid2002.131436

**Published:** 2014-02

**Authors:** Claudia Schulz, Kerstin Wernike, Martin Beer, Bernd Hoffmann

**Affiliations:** Friedrich-Loeffler-Institut, Greifswald-Insel Riems, Germany

**Keywords:** Schmallenberg virus, bovine semen, experimental infection, Germany, cattle, mice, viruses

**To the Editor:** The teratogenic Schmallenberg virus (SBV) (genus *Orthobunyavirus*) was detected in bovine semen in a recent German field study (*1*). Vector-borne transmission by *Culicoides* spp. biting midges is most common (*2*), but venereal transmission of SBV might contribute to the spread of this virus to previously unaffected regions. We investigated the infectivity of SBV RNA–positive semen by experimental subcutaneous injection of cattle and interferon α/β receptor–deficient (IFNAR^−/−^) mice (*3*).

Commercially produced semen straws with egg yolk–based diluent were used for the injection of 6- to 9-month-old heifers. The straws originated from 6 semen batches (quantification cycle [C_q_] values 26.4–36.4) collected from 6 bulls (designated A–C and E–G) during August and September 2012 (*1*). To increase the probability of SBV infection of injected cattle, 5 straws of semen (≈220 µL each) from 1 batch from an individual bull were pooled and diluted in minimal essential medium with antibiotics to 4 mL. Six cattle (C1–6) were subcutaneously inoculated, each with a pool from 1 of the 6 bulls. To investigate the infectivity of a single insemination dose (1 straw), 5 cattle (C7–C11) were subcutaneously injected with single straws from bull F that had been confirmed to contain infectious SBV. Serum samples were obtained on several days (Figure), and clinical signs and rectal body temperatures for the injected cattle were monitored daily.

**Figure:**
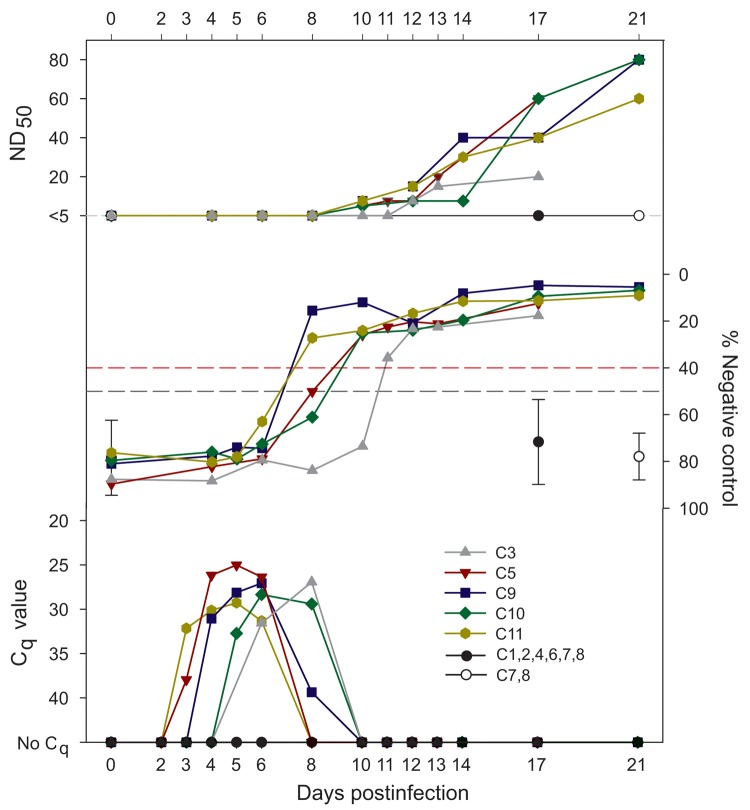
Detection of Schmallenberg virus (SBV) RNA and antibodies in serum of 5 of 11 cattle (C) injected with SBV RNA–positive bovine semen (quantification cycle [C_q_] values 26.4–36.4). Infectivity was measured by using reverse transcription PCR (C_q_), competitive ELISA (% negative control), and serum neutralization test (ND_50_, serum dilution that caused virus neutralization in 50% of the replicates). RNAemia and seroconversion occurred in C3, injected with pooled semen from bull C (C_q_ 34.2), and in C5 and C9–C11, injected with pooled or single semen straws from bull F (C_q_ 26.4). The onset of infection in cattle injected with semen from bull F varied between 3 and 5 days postinfection. Black lines indicate virologic and serologic results for cattle that remained uninfected. Dotted lines indicate positive (top) and negative (bottom) cutoff values; whiskers indicate SD of the mean.

In addition, 20 SBV RNA–positive semen batches (C_q_ 25.9 to 36.5) collected from 11 bulls (A–K) during August–November 2012 (*1*) were subcutaneously injected into 40 IFNAR^−/−^ mice (4–6 weeks old). For each batch, 2 mice were each injected with half of a semen straw (80–120 µL). All mice were monitored clinically and weighed daily. Samples of serum, liver, and spleen were harvested immediately after euthanasia at 22 days postinjection (dpi).

All serum samples and organ homogenates were tested for SBV RNA by using small segment–specific quantitative reverse transcription PCR (*4*). Serum samples were tested for SBV-specific antibodies by using the ID Screen Schmallenberg Virus Competition ELISA (IDvet, Montpellier, France), according to the manufacturer’s instructions; selected serum samples were also tested by neutralization test against an original SBV isolate from Germany, as described (*5*).

SBV infection was confirmed in 5 of 11 injected cattle: C3, C5, and C9–C11. SBV RNA (C_q_ 25.0–29.3) was first detected in serum at 3 to 6 dpi and persisted for 2–4 days. Seroconversion occurred at 8–12 dpi (Figure). None of the SBV-infected animals showed obvious clinical signs or fever; this finding is in accordance with reports of subclinical SBV infection in adult cattle (*5**–**7*). Samples from the other 6 cattle and all IFNAR^−/−^ mice had negative results (data not shown).

The 2 infectious semen batches contained moderate (C_q_ 26.4) or low (C_q_ 34.2) viral loads of SBV RNA, indicating that a high sensitivity is required for reliable SBV RNA detection in semen samples (*1*). The onset of SBV infection in the 3 animals injected with single semen straws ranged from 3 to 5 dpi, and not every straw was infectious, although biologic and technical replicates of straws from 1 semen batch showed similar PCR results (data not shown) (*1*). Possible explanations for differences in the infectivity of individual straws are that the viral RNA load of an SBV-containing straw does not necessarily correlate with infectivity or that the infectivity of 1 straw is lower than the minimal cattle infectious dose for SBV. Cattle might be more susceptible than IFNAR^−/−^ mice to infection, particularly when SBV titers are low or borderline (*7**, **8*). Therefore, we cannot exclude the possibility that the semen batches tested only in IFNAR^−/−^ mice might be infectious for cattle or that semen samples with higher SBV titers might be infectious in the mice.

We used subcutaneous injection of SBV RNA–positive semen to demonstrate infectivity because this transmission route has a high sensitivity for proving infectivity of SBV-containing samples (*7*). However, the possibility of intrauterine SBV infection of dams is unknown. Oro-nasal inoculation of 2 calves did not result in SBV infection of the animals (*5*), which suggests that mucosal in utero infection with SBV-containing semen is unlikely. In contrast, viremia was detected in most cows that were artificially inseminated and simultaneously inoculated in the uterus with cell culture–passaged Akabane virus, a teratogenic orthobunyavirus closely related to SBV (*9*). Intrauterine lesions caused by insemination or breeding might therefore increase the risk for SBV infection.

In conclusion, we demonstrated that SBV RNA–positive bovine semen could contain infectious SBV. However, the actual risk for transmission of SBV by insemination of dams with SBV-containing semen remains to be evaluated. Although SBV infection of the developing embryo is unlikely, venereal transmission would lead at worst to viremia of the dam, facilitating vector transmission. To prevent venereal SBV transmission, sensitive PCR testing of semen batches from SBV-infected bulls is the method of choice (*1*,*10*).

## References

[1] Hoffmann B, Schulz C, Beer M. First detection of Schmallenberg virus RNA in bovine semen, Germany, 2012. Vet Microbiol. 2013 Sept 12;pii:S0378-1135(13)00439-2. Epub ahead of print. 10.1080/00480169.2012.738403 24100006

[2] Conraths FJ, Peters M, Beer M. Schmallenberg virus, a novel orthobunyavirus infection in ruminants in Europe: potential global impact and preventive measures. N Z Vet J. 2013;61:63–7 . 10.1080/00480169.2012.738403 23215779

[3] Müller U, Steinhoff U, Reis LF, Hemmi S, Pavlovic J, Zinkernagel RM, Functional role of type I and type II interferons in antiviral defense. Science. 1994;264:1918–21 . 10.1126/science.8009221 8009221

[4] Bilk S, Schulze C, Fischer M, Beer M, Hlinak A, Hoffmann B. Organ distribution of Schmallenberg virus RNA in malformed newborns. Vet Microbiol. 2012;159:236–8 . 10.1016/j.vetmic.2012.03.035 22516190

[5] Wernike K, Eschbaumer M, Schirrmeier H, Blohm U, Breithaupt A, Hoffmann B, Oral exposure, reinfection and cellular immunity to Schmallenberg virus in cattle. Vet Microbiol. 2013;165:155–9 . 10.1016/j.vetmic.2013.01.040 23452751

[6] Hoffmann B, Scheuch M, Höper D, Jungblut R, Holsteg M, Schirrmeier H, Novel orthobunyavirus in cattle, Europe, 2011. Emerg Infect Dis. 2012;18:469–72 . 10.3201/eid1803.111905 22376991 PMC3309600

[7] Wernike K, Eschbaumer M, Breithaupt A, Hoffmann B, Beer M. Schmallenberg virus challenge models in cattle: infectious serum or culture-grown virus? Vet Res. 2012;43:84 . 10.1186/1297-9716-43-84 23231006 PMC3538505

[8] Wernike K, Kohn M, Conraths FJ, Werner D, Kameke D, Hechinger S, Transmission of Schmallenberg virus during winter, Germany. Emerg Infect Dis. 2013;19:1701–3 . 10.3201/eid1910.130622 24050688 PMC3810758

[9] Parsonson IM, Della-Porta AJ, Snowdon WA, O’Halloran ML. The consequences of infection of cattle with Akabane virus at the time of insemination. J Comp Pathol. 1981;91:611–9 . 10.1016/0021-9975(81)90090-6 6798085

[10] van Oirschot JT. Bovine herpesvirus 1 in semen of bulls and the risk of transmission: a brief review. Vet Q. 1995;17:29–33 . 10.1080/01652176.1995.9694526 7610554

